# Laparoscopic surgery for Crohn’s disease: a meta-analysis of perioperative complications and long term outcomes compared with open surgery

**DOI:** 10.1186/1471-2482-13-14

**Published:** 2013-05-24

**Authors:** Sunil V Patel, Sanjay VB Patel, Sreeram V Ramagopalan, Michael C Ott

**Affiliations:** 1 Divisions of General Surgery, The University of Western Ontario London, Ontario, ON, Canada; 2 Department of Epidemiology, London School of Hygiene and Tropical Medicine, London, United Kingdom; 3 Wellcome Trust Centre for Human Genetics, University of Oxford, Oxford, United Kingdom; 4 Nuffield Department of Clinical Neurosciences, University of Oxford, Oxford, United Kingdom

**Keywords:** Laparoscopy, Crohn’s disease, Perioperative complications, Surgical recurrence, Hernia, Small bowel obstruction

## Abstract

**Background:**

Previous meta-analyses have had conflicting conclusions regarding the differences between laparoscopic and open techniques in patients with Crohn’s Disease. The objective of this meta-analysis was to compare outcomes in patients with Crohn’s disease undergoing laparoscopic or open surgical resection.

**Methods:**

A literature search of EMBASE, MEDLINE, The Cochrane Central Register of Controlled Trials and the US National Institute of Health’s Clinical Trials Registry was completed. Randomized clinical trials and non-randomized comparative studies were included if laparoscopic and open surgical resections were compared. Primary outcomes assessed included perioperative complications, recurrence requiring surgery, small bowel obstruction and incisional hernia.

**Results:**

34 studies were included in the analysis, and represented 2,519 patients. Pooled analysis showed reduced perioperative complications in patients undergoing laparoscopic resection vs. open resection (Risk Ratio 0.71, 95% CI 0.58 – 0.86, P = 0.001). There was no evidence of a difference in the rate of surgical recurrence (Rate Ratio 0.78, 95% CI 0.54 – 1.11, P = 0.17) or small bowel obstruction (Rate Ratio 0.63, 95% CI 0.28 – 1.45, P = 0.28) between techniques. There was evidence of a decrease in incisional hernia following laparoscopic surgery (Rate Ratio 0.24, 95% CI 0.07 – 0.82, P = 0.02).

**Conclusions:**

This is the largest review in this topic. The results of this analysis are based primarily on non-randomized studies and thus have significant limitations in regards to selection bias, confounding, lack of blinding and potential publication bias. Although we found evidence of decreased perioperative complications and incisional hernia in the laparoscopic group, further randomized controlled trials, with adequate follow up, are needed before strong recommendations can be made.

## Background

Despite many advances in the medical management of Crohn’s disease, there is still a significant risk of surgical resection during the lifetime of a patient. Over 80% of patients diagnosed with primary ileocolic Crohn’s disease have a surgical resection within 10 years of their diagnosis [1]. In patients undergoing initial surgical resection, over one quarter will require subsequent surgical resections for recurrent disease within five years of the initial resection [1]. Bernell et al., found that the median age of the first surgical resection in Crohn’s disease is in the third decade. Given the young age of the patient population and the recurrent nature of the disease process, this patient population seems ideally suited for a laparoscopic approach to resection.

A Cochrane Review [2] on this topic was recently published. It focused on randomized controlled trials only. This review included 120 patients from 2 trials, and found no significant differences in perioperative complications or long-term outcomes. The pooled results were likely underpowered to detect a difference in the long term outcomes. For recurrences requiring reoperation, a sample size of over 2800 patients per group would be needed to detect the difference seen in this review (for a power of 80% and an α of 0.05).

Previous meta-analyses [3-6] have been completed comparing laparoscopic and open surgery for Crohn’s disease. The focus of these analyses was primarily on perioperative factors such as operating time, length of stay, return to diet and time to bowel movement. There were conflicting conclusions on the effects of laparoscopic surgery on perioperative complications and in the rate of recurrence. Only one of the analyses assessed the effect of small bowel obstruction [3] and found a benefit to laparoscopic surgery.

Since the publication of the most recent meta-analysis, there have been 13 publications exploring both perioperative complications and long-term outcomes in patients undergoing laparoscopic resection of Crohn’s disease. This new data may help to clarify the inconclusive findings of prior meta-analyses, and warrants an up to date analysis.

This study assesses the risk of perioperative complications and long-term outcomes in patients undergoing resection for Crohn’s disease either by laparoscopic or open surgery.

## Methods

This Meta-Analysis adheres to the MOOSE reporting guidelines [7].

### Eligibility criteria

Both randomized clinical trials and non-randomized studies were included. The population of interest were patients undergoing bowel resection for Crohn’s disease. The intervention was laparoscopic surgical resection (including hand assisted laparoscopic resection), compared with open surgical resection.

Primary outcomes included both perioperative complications and long-term outcomes. Perioperative complications were defined as complications occurring within 30 days of surgery including wound infection, urinary tract infections, respiratory complications, anastomotic leak, intra abdominal abscess, bowel obstruction or prolonged ileus and reoperation for any reason. Long-term outcomes included recurrence of Crohn’s disease requiring surgical intervention, small bowel obstruction and incisional hernia.

Publications comparing laparoscopic to open colorectal surgery were also included if data for patients undergoing resection for Crohn’s disease could be extracted separately. Studies were excluded if there was no open surgery control group for comparison. Only the most recent publication data was included for analysis if there was duplication of patient data across multiple studies.

### Information sources and search

The search was completed by two of the authors (SVP, SVBP), with input for search strategy from the institutions librarian. A search was completed in December, 2011 using EMBASE (1980 – 2011), MEDLINE (1948 – 2011), The Cochrane Central Register of Controlled Trials and the US National Institute of Health’s Clinical Trials Registry. Bibliographies of related studies were searched. Search terms included “Crohn’s Disease”, “inflammatory bowel disease”, “Laparoscopy” and “laparoscopic surgery”. Terms were combined with Boolean expressions to limit the search. The study was not limited to English language journals. Non-english language articles were translated into English.

Two independent reviewers assessed titles and abstracts for relevant articles. Full text review was performed on identified studies. Both reviewers assessed each study, to determine if it met the inclusion criteria. The kappa statistic was used to assess inter-rater agreement.

Data was extracted by two independent reviewers using standardized forms. Study details, patient characteristics, follow up periods and outcomes were extracted.

### Risk of bias assessment

Study quality was assessed using two methods. Non-randomized comparative trials were assessed using the Newcastle – Ottawa quality assessment scale, [8] as recommended in the Cochrane Handbook [9]. This scale assigns a star rating based on pre-specified criteria. A maximum of one star can be attained for each category, except comparability which has a maximum of 2 stars. The more stars a study obtains, the higher the quality. Randomized controlled trials were assessed as discussed in the Cochrane Handbook [9].

### Statistical analysis

Data was analyzed using STATA 12 (StataCorp LP, College Station Texas, 2011). For short- term complications, risk ratios and 95% confidence intervals were calculated. Rate ratios and 95% confidence intervals, using patient years, were calculated for long-term outcomes. Using rates instead of risk allows for comparison between groups with unequal follow up duration.

The fixed effect model using the inverse variance method was used for these calculations. Risk difference and 95% confidence intervals were calculated for continuous variables, using the fixed effect model with the inverse variance method. A fixed effect model was selected because of the large number of trials and large variation in study size. This allows for larger trials to be weighted more strongly in the pooled estimate. I^2^ values were used to determine heterogeneity between studies.

Publication bias was assessed using funnel plots. Statistical test for funnel plot asymmetry was completed as recommended in the Cochrane Handbook.

### Additional analysis

*A priori* subgroup analysis was planned to assess differences in perioperative complications between randomized controlled trials and non-randomized comparative trials. Subgroup analysis was also planned to determine if the type of resection affected the long term outcomes. The test of interaction was used to assess heterogeneity between subgroups.

Sensitivity analysis was completed for rates of long-term outcomes. For our analysis, rate ratios were calculated using studies with known follow up duration in both groups. A sensitivity analysis was completed, including studies with incomplete follow up data, to assess the effect these studies had on the rate ratio. In this analysis, it was assumed that follow up duration was the same between groups and equal to the duration given for the entire study population. The test of interaction was used to assess the heterogeneity between these rate ratios.

## Results

**Figure 1:**
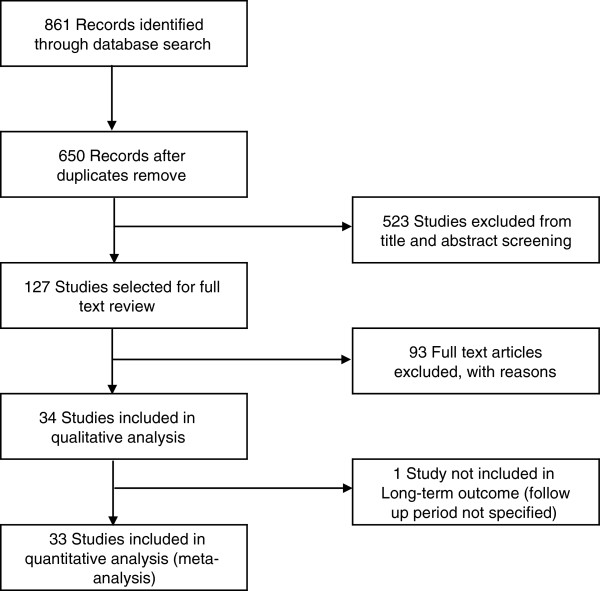
Flow Diagram of search results, and trial inclusion.

A literature search found 861 articles. After eliminating duplicates and identifying relevant studies, 127 articles were selected for full text review. Of these, 34 articles were included in the study (Figure 1). A total of 2,519 patients were included in our analysis. Two independent reviewers assessed articles for inclusion. The weighted kappa statistic was 0.92, which indicated very good agreement between the two reviewers. Disagreements were resolved through discussion and consensus.

A summary of included articles can be found in Table 1. Included studies were published from 1997 – 2011, and included both randomized controlled trials and non-randomized studies. The number of patients in these studies ranged from 13 – 456. Weighted Mean Differences in age, BMI, length of disease, length of stay, and time to diet, flatus and bowel movement can be found in Table 2. Risk ratio of preoperative steroid use and previous surgery can also be found in Table 2. Laparoscopic patients were younger (P = 0.03), while other characteristics were similar between groups. Shorter length of stay, earlier return to solid diet, time to flatus and time to bowel movement were seen in the laparoscopic group.

**Table 1:** **Summary of included studies: NR non-randomized, RCT randomized control trial, IC Ileocecal, PO perioperative complications, LT long term outcomes, **^**a **^**Median displayed**

**Study**	**Patient No.**	**Methods**	**Participants**	**Intervention**	**Outcomes**	**Long term follow up (Mean, months)**	**Notes**
Alabaz et al. 2000 [10]	74	NR	CD patients	IC resection	PO, LT	Study mean = 30	
Allesandroni 2010 [11]	200	NR	CD patients	IC resection	PO, LT	Lap = 52; Open = 60	
Bemelman et al. 2000 [12]	78	NR	CD patients	IC resection	PO		
Benoist et al. 2003 [13]	56	NR	CD patients	IC resection	PO		Cases and controls matched
Bergamaschi et al. 2003 [14]	92	NR	CD patients	IC resection	PO, LT	Lap = 60; Open = 60	
Broquet et al. 2010 [15]	62	NR	CD patients	IC resection	PO		Patients with at least 1 previous resection
Courtney et al. 2011 [16]	13	NR	Adolescent patients with IBD	Any bowel resection and anastomosis	PO		
da Luz Moreira et al. 2007 [17]	54	NR	Crohn’s colitis patients	Colectomy for Crohn’s colitis	PO, LT	Lap = 12; Open = 40^a^	Cases and controls matched
Diamond et al. 2001 [18]	23	NR	Adolescent patients with CD	IC resection	PO		
Duepree et al. 2002 [19]	45	NR	CD patients	IC resection	PO		
Dunker et al. 1998 [20]	22	NR	CD patients	IC resection	PO,		
El Gazaaz et al. 2010 [21]	456	NR	Patients undergoing bowel anastomosis	Any bowel resection and anastomosis	Anastamotic leak rate		Cases and controls matched
Eshuis et al. 2008 [22]	71	NR	CD patients	IC resection	LT	Lap = 104; Open = 103^a^	Long-term follow up to study by Bemelman 2000
Eshuis et al. 2010 [23]	55	RCT	CD patients	IC resection	LT	Lap = 78; Open = 82^a^	Follow up report on the RCT completed by Maartense et al. 2006
Fichera et al. 2007 [24]	146	NR	CD patients	IC resection	PO, LT	Lap = 26, Open = 15	
Huilgol et al. 2004 [25]	40	NR	CD patients	IC resection	PO		
Kishi et al. 2000 [26]	35	NR	Stenotic lesions in CD patients	IC resection	PO		
Lauro et al. 2004 [27]	51	NR	CD patients	Any bowel resection and anastomosis	PO		
Lowney et al. 2006 [28]	113	NR	CD patients	IC resection	PO, LT	Lap = 60; Open = 81	
Luan et al. 2000 [29]	40	NR	CD patients	Any bowel resection and anastomosis	PO		
Maartense et al. 2006 [30]	60	RCT	CD patients	IC resection	PO		
Milsom et al. 2001 [31]	60	RCT	CD patients	IC resection	PO		
Msika et al. 2001 [32]	46	NR	CD patients	Any bowel resection and anastomosis	PO		
Nakajima et al. 2010 [33]	38	NR	Crohn’s colitis	Colectomy or subtotal colectomy	PO		Hand assisted and laparoscopic assisted groups analyzed together
Shore et al. 2003 [34]	40	NR	CD patients	IC resection	PO		
Sica et al. 2008 [35]	28	NR	CD patients	IC resection	PO		
Stocchi et al. 2008 [36]	56	RCT	CD patients	IC resection	LT	Lap = 120; Open = 132	Follow up report on the RCT completed by Milsom et al. 2001
Tabet et al. 2001 [37]	61	NR	CD patients	Any bowel resection and anastomosis	PO, LT	Lap = 39; Open = 42	
Tanaka et al. 2008 [38]	48	NR	CD patients	Any bowel resection and anastomosis	PO, LT	Unknown	
Thaler et al. 2005 [39]	37	NR	CD patients	IC resection	LT		
Uchikoshi et al. 2004 [40]	43	NR	Recurrent CD	Bowel resection and anastomosis	PO, LT	Lap = 29.6, Open = 71.9	Hand assisted and laparoscopic assited groups analyed together
Umanskiy et al. 2010 [41]	125	NR	Crohn’s colitis patients	Colectomy for Crohn’s colitis	PO, LT	Lap = 20; Open = 29	
Von Allmen et al. 2003 [42]	28	NR	CD patients	Any bowel resection and anastomosis	PO		
Wu et al. 1997 [43]	123	NR	CD patients	IC resection	PO		

*NR* Non-randomized, *RCT* randomized control trial, *IC* Ileocecal, *PO* perioperative complications, *LT* Long Term Outcomes, ^a^ Median displayed.

**Table 2:** **Differences between laparoscopic and open surgery patients, **^**a **^**Mean difference **^**b **^**Risk ratio**

	**No. studies**	**Mean difference or risk ratio (95% CI)**	**P Value**
Age, yr	24 (n = 2011)	−1.41 (−2.71 to −0.11)^a^	0.03
BMI, kg/m^2^	14 (n = 1359)	−0.65 (−1.94 to 0.65)^a^	0.32
Length of disease, years	14 (n = 974)	−0.94 (−3.02 to 1.15)^a^	0.38
Previous surgery	16 (n = 1201)	0.80 (0.64 to 1.01)^b^	0.06
Preoperative steroids	13 (n = 921)	1.06 (0.95 to 1.17)^b^	0.33
Length of stay, days	17 (n = 1005)	−2.24 (−2.50 to −1.98)^a^	<0.001
Return to solid diet, days	9 (n = 442)	−1.29 (−1.59 to −0.98)^a^	<0.001
Time to flatus, days	8 (n = 465)	−0.80 (−1.05 to −0.55)^a^	<0.001
Time to bowel movement, days	6 (n = 378)	−0.68 (−0.94 to −0.43)^a^	<0.001

There were four articles which included data from two randomized controlled trials [23,30,31,36]. The studies by Eshius [23] and Stocchi [36] included long-term outcomes from the initial studies by Maartense [30] and Milsom [31], respectively. The risk of bias of these studies can be found in Table 3. In both cases the risk of bias was deemed to be high. Neither study had adequate blinding of study personnel or patients. As well, in the study by Milsom et al., the decision for randomization did not occur until after a diagnostic laparoscopy was completed and the patient deemed appropriate for laparoscopic resection.

**Table 3:** Risk of bias of randomized controlled trials

**Study**	**Randomization**	**Allocation concealment**	**Blinding**	**Incomplete outcome data**	**Selective reporting**	**Other potential sources of bias**	**Risk of bias**
Milsom 2001 [31]	Unclear method of randomization	Unclear: not discussed	High risk: unable to blind due to type of intervention	Low risk	Low risk	Unclear risk: not intention to treat analysis. Patients randomized after laparoscopy	High
Maartense 2006 [30]	Unclear method of randomization	Low risk: sealed envelopes	High risk: unable to blind due to type of intervention	Low risk	Low risk	None	High

The quality assessment of the non-randomized trials can be found in Table 4. Thirty observational trials [10-22,24-29,32-35,37-43] were included in this analysis. Twenty four of the 30 trials (80%) used consecutive patients over a specified time period in selecting laparoscopic patients. These are likely representative of Crohn’s patients requiring surgical intervention. The other six studies did not use consecutive patients, and did not describe how the included laparoscopic patients were selected. Twenty one of the studies (70%) selected open surgical patients from the same population as the laparoscopic patients. The other 9 studies selected the open surgical patients from historic periods. The laparoscopic and open groups were comparable for at least age and gender in 20 studies (67%). Three of the studies matched the open group to the laparoscopic group on several different factors, including age, gender, body mass index, disease severity or steroid use. Adequate follow up duration was seen in all studies looking at short term outcomes. Of the 12 observation studies looking at long term outcomes [10,11,14,17,22,24,28,37-41], 3 studies [17,24,41] had less than 2 years of follow up in one of the groups, while one [38] did not specify the follow up duration at all.

**Table 4:** Quality assessment of non-randomized controlled trials

	**Selection**					**Outcome**		
**Study**	**Representativeness of the exposed cohort**	**Selection of the non exposed cohort**	**Ascertainment of exposure**	**Outcome(s) absent at start of study**	**Comparability of cohorts**	**Assessment of outcome**	**Adequate follow up time**	**Adequacy of follow up**
Alabaz 2000 [10]	*	*	*	*		*	*	*
Allesandroni 2010 [11]	*		*	*	*	*	*	*
Bemelman 2000 [12]	*		*	*	*	*	*	*
Benoist 2003 [13]	*		*	*	**	*	*	*
Bergamaschi 2003 [14]	*		*	*	*	*	*	*
Broquet 2010 [15]	*	*	*	*	*	*	*	*
Courtney 2011 [16]	*		*	*		*	*	*
da Luz Moreira 2007 [17]	*	*	*	*	**	*		*
Diamond 2001 [18]	*	*	*	*	*	*	*	*
Duepree 2002 [19]	*	*	*	*		*	*	*
Dunker 1998 [20]		*	*	*		*	*	*
El Gazaaz 2010 [21]	*	*	*	*	**	*	*	*
Eshuis 2008 [22]	*	*	*	*	*	*	*	*
Fichera 2007 [24]	*	*	*	*		*		*
Huilgol 2004 [25]	*		*	*		*	*	*
Kishi 2000 [26]	*	*	*	*		*	*	*
Lauro 2004 [27]	*	*	*	*	*	*	*	*
Lowney 2006 [28]			*	*	*	*	*	*
Luan 2000 [29]			*	*		*	*	*
Msika 2001 [32]	*	*	*	*	*	*	*	*
Nakajima 2010 [33]	*	*	*	*		*	*	*
Shore 2003 [34]		*	*	*	*	*	*	*
Sica 2008 [35]	*	*	*	*	*	*	*	*
Tabet 2001 [37]	*	*	*	*	*	*	*	*
Tanaka 2008 [38]	*	*	*	*	*	*		*
Thaler 2005 [39]		*	*	*	*	*	*	*
Uchikoshi 2004 [40]			*	*	*	*	*	*
Umanskiy 2010 [41]	*	*	*	*	*	*		*
Von Allmen 2003 [42]	*	*	*	*	*	*	*	*
Wu 1997 [43]	*	*	*	*		*	*	*

A study can be awarded a maximum of 1 star (*) in each of the selection and outcome categories, and a maximum of 2 stars (**) in thecomparability section.

Two studies [33,40] included patients undergoing both hand assisted laparoscopic surgery and laparoscopic assisted surgery. These two groups were analyzed together in the laparoscopic surgery group. All other studies compared laparoscopic surgery to open surgery.

The majority of studies looked at patients undergoing ileocecal resection only. Nine studies [16,21,27,29,32,37,38,40],[42] included patients with any bowel resection. These resections included ileocecal resection, other small bowel resections, segmental colectomy and subtotal colectomy. Three studies [17,33,41] (20, 32, 39) looked exclusively at patients undergoing colectomy for Crohn’s colitis.

The study by El-Gazaaz et al. [21] looked at patients undergoing bowel resection and anastomosis. These included colorectal cancer patients, Crohn’s disease patients and patients with diverticular disease. The primary outcome in this study was anastomotic leak rate and Crohn’s disease patients were analyzed independently, and matched to an open surgery control group. One study [16] included both Crohn’s disease and ulcerative colitis patients, and included data for the subset of Crohn’s disease patients. All other studies looked exclusively at Crohn’s disease patients.

### Perioperative complications

**Figure 2:**
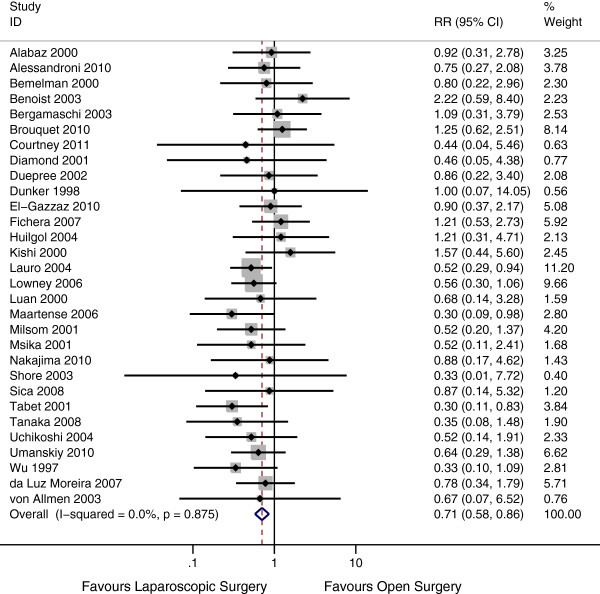
Forest plot of perioperative complications, risk ratio 0.71 (95% CI 0.58 – 0.86, P = 0.001).

**Figure 3:**
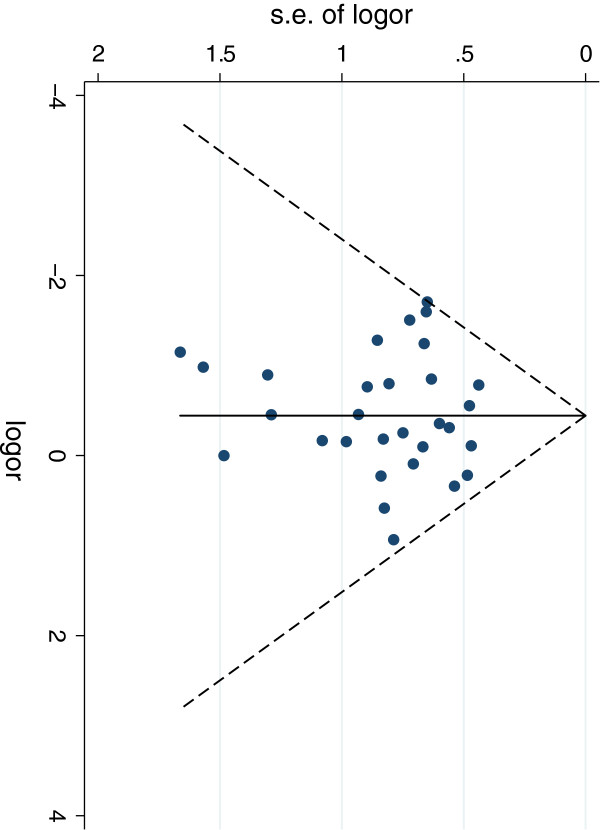
**Funnel plot from perioperative complications.** Test for assymetry, P = 0.75.

Thirty studies [10-21,24-35,37,38,40-43] addressed the perioperative complication risk in the two groups. This included 1079 laparoscopic patients and 1221 open surgery patients. The perioperative complication risk in the laparoscopic group was 12% compared with 18% in the open group. There is very strong evidence of a reduced risk of perioperative complications in the laparoscopic group (Risk Ratio 0.71, 95% CI 0.58 – 0.86, P = 0.001). There was statistical homogeneity in this outcome, with an I^2^ of 0% (P = 0.87) (Figure 2). Publication bias was assessed by funnel plot (Figure 3) There was no evidence of assymetry seen in the funnel plot (P = 0.75).

A subgroup analysis was completed evaluating the two randomized controlled trials. This subgroup showed a significant decrease in perioperative complications in laparoscopic surgery patients compared with open surgery patients (Risk Ratio 0.42; 95% CI 0.20 – 0.88; p = 0.02). The test of interaction between this subgroup and the non-randomized trials showed no evidence of a subgroup effect (p = 0.16), indicating that the findings between the randomized controlled trials and non-randomized trials were compatible.

All assessed complications were consistent with a reduction in the laparoscopic group. Due to the lack of power, none of these measures reached significance. We found no evidence of a difference in risk of intraabdominal abscess (P = 0.19), risk of prolonged ileus or perioperative bowel obstruction (P = 0.49), risk of wound infection (P = 0.43), risk of anastomotic leak rate (P = 1.00), risk of 30 day re-operation rate (P = 0.32), risk of urinary tract infection (P = 0.46) or risk of respiratory complications (P = 0.19) (Table 5).

**Table 5:** Pooled risk ratios of complications in Crohn’s patients undergoing resection by laparoscopic or open technique

**Perioperative complication**	**Number of studies**	**Total number of patients**	**Laparoscopic**	**Open surgery**	**Risk ratio**	**95% Confidence interval**	**P Value**	**I2**
All	30	2300	12.00%	17.90%	0.71	0.58 - 0.86	0.001	0%
Wound infection	25	1670	5.80%	6.10%	0.86	0.60 - 1.25	0.43	0%
Prolonged Ileus/ bowel obstruction	14	1012	3.90%	4.70%	0.83	0.48 – 1.42	0.49	0%
Respiratory complication	11	825	0.80%	2.50%	0.57	0.25 – 1.33	0.19	0%
Urinary tract infection	5	367	1.90%	3.30%	0.65	0.21 – 2.02	0.46	0%
Anastamotic leak	12	1261	2.70%	2.70%	1	0.55 – 1.82	1	0%
Intraabdominal abscess	15	1121	2.70%	4.40%	0.69	0.39 – 1.20	0.19	0%
<30 day reoperation	13	917	2.40%	4.00%	0.72	0.37 – 1.38	0.32	0%

### Long –term outcomes

Long-term outcomes included surgical recurrence, small bowel obstruction and incisional hernia. A total of 14 studies [10,11,14,17,22-24,28,36-41] assessed at least one of these outcomes. The duration of follow up was variable between studies and ranged from 12 to 132 months.

To negate the variability in the follow up period between groups, rates of long term outcomes were assessed based on person years of follow up. Follow up duration by intervention was included in 11 studies, and incomplete in 3 studies. The study by Tanaka et al. [38] did not specifically address the duration of the follow up period and was excluded from further analysis. The studies by Alabaz et al. [10] and Thaler et al. [39] did not specify follow up duration by individual group, but instead gave the average follow up of all patients.

Surgical recurrence was discussed in 12 studies, 10 of which included follow up duration by group. These 10 studies represented a total of 4,323 person years. Only two of the studies [22,28] discussed the use of chemoprophylaxis post operatively to prevent recurrence. Neither study found a difference in rates of chemoprophylaxis between groups.

**Figure 4:**
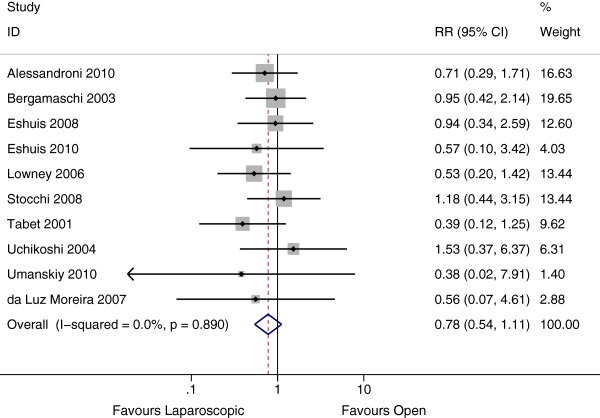
**Forest plot of surgical recurrence, rate ratio 0.78, (95% CI 0.54 – 1.11, P = 0.17).**

The rate of surgical recurrence in the laparoscopic group was 25 per 1000 person years compared with 34 per 1000 person years in the open group. This difference was not significant (Rate Ratio 0.78, 95% CI 0.54 – 1.11, P = 0.17, I^2^ = 0%) (Figure 4). There was no evidence of publication bias from the funnel plot (P = 0.65).

Including the studies by Alabaz et al. and Thaler et al., showed a similar rate ratio with no significant difference between these estimates (P = 1.00). Subgroup analysis by type of resection was performed. There was no evidence of a difference in recurrence rates between patients undergoing ileocolic resection, colectomy or other resection (P = 0.45).

**Figure 5:**
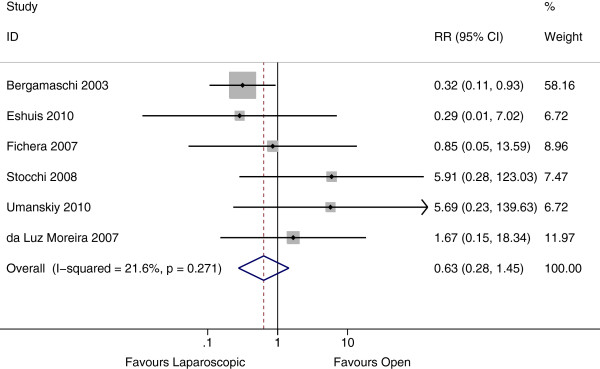
Forest Plot of small bowel obstruction, rate ratio 0.63 (95% CI 0.28 – 1.45, P = 0.28).

Small bowel obstruction was assessed in 7 studies, 6 of which included follow up duration by group. These 6 studies represented a total of 1991 person years. The rate of small bowel obstruction was 10 per 1000 person years in the laparoscopic group compared with 19 per 1000 person years in the open group. There was no evidence of a difference between groups (Rate Ratio 0.63, 95% CI 0.28 – 1.45, P = 0.28, I^2^ = 26%) (Figure 5). No publication bias was appreciated from the funnel plot (P = 0.15). Including the study by Alabaz et al. in this analysis showed a similar rate ratio (P = 0.69).

**Figure 6:**
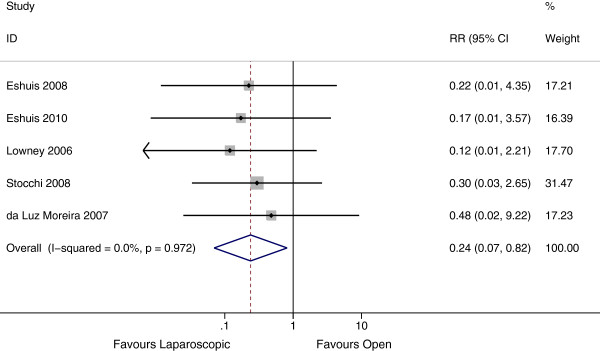
Forest plot of incisional hernia, rate ratio 0.24 (95% CI 0.07 – 0.82, P =0.02).

Incisional hernia was evaluated in 6 studies, 5 of which included follow up by group. There were a total of 2329 person years in these 5 studies. The rate of incisional hernia in the laparoscopic group was 1 per 1000 person years, compared with 12 per 1000 person years in the open group (Rate Ratio 0.24, 95% CI 0.07 – 0.82, P = 0.02, I^2^ = 0%) (Figure 6). Including the study by Thaler et al. in the analysis, showed a similar effect estimate, with no difference between estimates (P = 0.91).

## Discussion

An updated meta-analysis of laparoscopic surgery in Crohn’s disease was performed. This analysis includes more than double the number of trials of previous meta-analyses and to our knowledge represents the largest and most complete collection of published data on this topic.

Previous meta-analyses have had conflicting results regarding the benefits of laparoscopic surgery in reducing perioperative complications. The studies by Dasari et al. [2], Polle et al. [6] and Tilney et al. [5] found no evidence of a difference in perioperative complications while Rosman et al. [3] and Tan et al. [4] did find a significant difference. Through the inclusion of an additional 13 studies and over 1300 patients compared with the previous meta-analyses, we were able to show evidence of a decrease in total perioperative complications in the laparoscopic group. In all assessed complications, the Risk Ratio favoured laparoscopic surgery, but due to the imprecision in the estimate, this effect did not reach statistical significance. The lack of imprecision is related to the pooled estimate being underpowered to detect a difference in individual complications. Due to the infrequency of complications (~5% per individual complication) seen in the open group, a study size of close to 14,000 subjects would be required to detect a 20% reduction in complication risk, with 80% power and 5% significance.

Whilst the majority of the trials were observational and have their clear limitations as discussed below, a subgroup analysis of RCTs also provided evidence of benefit of laproscopic surgery. The summary effect estimate of the RCTs was greater than that of all studies combined further highlighting the imprecision of the currently available data.

We found no evidence of a difference in rates of surgical recurrence or rates of small bowel obstruction, after pooled analysis. A previous meta-analysis [3] found a significant reduction in surgical recurrence with laparoscopic surgery (OR 0.51, 95% CI 0.27 – 0.80). This analysis included 6 studies. [10,14,32,34,36,37] Our study included an additional 7 studies [11,17,22,23,28,40,41] and excluded 2 of the trials used in the previous meta-analysis because [32,34] neither of these two trials described surgical recurrence as an outcome. The prior result was limited by not accounting for the difference in follow up period between groups. Our results were based on rates, which allowed us to compare surgical recurrence between laparoscopic and open groups directly, and more accurately than the previous review.

### Strengths and limitations

The strengths of this review include the thorough search of eligible studies, using explicit eligibility criteria, and the assessment of study quality. The breadth of this search allowed inclusion of over 30 studies and 2,510 patients, which corresponds to the largest, most comprehensive meta-analysis in this subject to date. We were also able to compare our results to those reported in previous meta-analyses.

Limitations of this analysis include the large number of non-randomized studies and limited number of randomized controlled trials. Unfortunately, only 2 randomized controlled trials exist, and included only 120 patients in total. These RCTs were also of poor quality with high risk of bias.

Although many of the included observational studies had similar baseline characteristics in the two groups, we did see a younger age in the laparoscopic group. In addition, these observation studies likely suffered from selection bias. Choosing healthier patients, with lower BMI and less comorbidities was likely more frequent in the laparoscopic group. These types of patients would be less likely to suffer post operative complications and incisional hernias.

Additionally, confounders (both known and unknown) are likely to exist within these observation studies. None of the included studies attempted to adjust for known confounders (such as immune suppression, age, comorbidities, BMI). Adjusting for these variable may have resulted in a different pooled result. The use of chemoprophylaxis for preventing recurrence was discussed in only two of the 12 trials evaluating surgical recurrence, and differences may have an impacted our findings on recurrence.

A third limitation of the included observational studies, is the likely bias of the authors. In many cases, the authors were advocates of the laparoscopic technique and were more experienced in this technique than the majority of surgeons treating this condition. This may skew their results towards favouring laparoscopic techniques. Non-experts may not have the same results as in the publications.

There was also no attempt at blinding patients in the included study. With the adoption of laparoscopic surgery as state of the art, there is a strong possibility of placebo effect.

## Conclusions

Based primarily on non-randomized trials, laparoscopic surgery appears to reduce the risk of perioperative complications and the rate of incisional hernia in patients with Crohn’s disease. There was no significant difference in long term outcomes between laparoscopic and open surgery, in terms of surgical recurrence or small bowel obstruction.

There is clearly a continued interest in assessing and reporting experience with laparoscopic surgery in Crohn’s disease. We have shown that most of the evidence of outcomes surrounding this technique is based on non-randomized studies, which suffer from selection bias, detection bias, confounding and a lack of blinding. Laparoscopic technique has been widely adopted despite the lack of evidence. As such, we would recommend that any further studies in this area address current limitations and be appropriately powered randomized controlled trials, with adequate follow up duration.

## Competing interests

The author’s declare no competing interests in the publication of this manuscript.

## Authors’ contributions

SVP, MCO designed the study. SVP, SVBP completed the search and data extraction. SVP, SVBP, MCO, SVR completed the introduction, analysis and discussion.

## Pre-publication history

The pre-publication history for this paper can be accessed here:

http://www.biomedcentral.com/1471-2482/13/14/prepub
